# Impact of ALDH1A1 Expression in Intrahepatic Cholangiocellular Carcinoma

**DOI:** 10.7150/jca.99944

**Published:** 2025-01-01

**Authors:** Konrad Kurowski, Melanie Föll, Tilman Werner, Oliver Schilling, Martin Werner, Stefan Fichtner-Feigl, Bertram Bengsch, Peter Bronsert, Philipp Anton Holzner, Sylvia Timme

**Affiliations:** 1 Institute for Surgical Pathology, Medical Center - University of Freiburg, Faculty of Medicine, University of Freiburg, Germany.; 2 Core Facility Histopathology and Digital Pathology Freiburg, Medical Center - University of Freiburg, Germany.; 3 Faculty of Biology, University of Freiburg, Germany.; 4 Spemann Graduate School for Biology and Medicine (SGBM), Freiburg, Germany.; 5 Tumorbank Comprehensive Cancer Center Freiburg, Medical Center - University of Freiburg, Germany.; 6 German Cancer Consortium (DKTK) and German Cancer Research Center (DKFZ), Heidelberg, Germany.; 7 Department of General and Visceral Surgery, Medical Center - University of Freiburg, Faculty of Medicine, University of Freiburg, Germany.; 8 Faculty of Medicine, Clinic for Internal Medicine II, Gastroenterology, Hepatology, Endocrinology, and Infectious Disease, University Medical Center Freiburg, Freiburg, Germany.

## Abstract

**Background:** Intrahepatic cholangiocarcinoma (iCC) is a rare malignant liver tumor with limited therapeutic advancements. Despite its increasing global incidence knowledge of treatment options remains stagnant, leading to poor five-year patient survival rates and high recurrence post-surgery. ALDH1A1, a member of the ALDH superfamily, is associated with cancer stem cells and has conflicting reports regarding its prognostic role in iCC.

This retrospective study analyzed 69 iCC patient samples from University Hospital Freiburg. Tissue microarrays (TMAs) were constructed, and ALDH1A1 expression was immunohistochemically assessed using machine learning algorithms. Script-based Survival analysis employed Kaplan-Meier curves, log-rank tests, and Cox Proportional Hazards Models.

ALDH1A1 overexpression, both in tumor and stromal cells, correlates with favorable overall survival in iCC. Gender-specific analyses indicate a more pronounced effect in females. These findings suggest ALDH1A1 as a potential prognostic biomarker in iCC, warranting further validation in larger cohorts and exploration as a therapeutic target.

## Introduction

Intrahepatic cholangiocarcinoma (iCC) is the second most common primary malignant tumor of the liver after hepatocellular carcinoma (HCC). Overall, representing about 15% of all primary liver tumors, iCC is a rare tumor entity, accounting for only 3% of all malignant gastrointestinal tumors 1,2. Nevertheless, the incidence of iCCs is increasing worldwide, but interestingly, knowledge about therapeutic treatment options is almost standing still. This might be attributed to the relatively limited foundational knowledge of iCC in comparison to other entities. The stagnation of knowledge is best reflected by the dismal five-year patient overall survival of up to of 20% and the high recurrence rates after surgical resection 3-5.

Intrahepatic cholangiocarcinoma derives from the interlobular bile ducts 6. Considering the histopathological growth pattern, iCCs form tumor masses, are periductal-infiltrating, or intraductal-growing 1,7.

Risk factors for iCC comprise hepatitis B/C infection, parasitic liver infections, liver cirrhosis, cholestasis, diabetes mellitus type II, obesity, alcohol consumption, smoking, asbestosis, and exposure to organic solvents 8-10.

The aldehyde dehydrogenase (ALDH) superfamily comprises a nicotinamide-adenine dinucleotide phosphate-positive (NAD-P+) dependent enzyme. ALDH catalyzes the oxidation of aldehydes to their corresponding carboxylic acids and converts retinol into retinoic acid 11. Subcellularly, ALDH is predominantly found within the cytosol but can also be detected within the mitochondria, the endoplasmic reticulum, and / or the nucleus 12.

Within the ALDH superfamily, ALDH1A1 has been described as a marker for cancer stem cells (CSCs), first reported by Ginestier *et al.* 13. Ginestier *et al.* demonstrated that in breast cancer elevated ALDH1 levels correlated with poor patient overall survival and an increased tumor-initiating ability *in vivo*. Subsequent studies not only supported the findings of Ginestier *et al.* 14-17 but also depicted biologically relevant ALDH1A1-triggered mechanisms such as tumor growth, drug - 18-20 and irradiation resistance 21,22.

Nevertheless, more and more publications indicate a protective biological function of ALDH1A1 within the tumor, as well as within the surrounding tumor stroma 23-28.

Considering iCC, only a few publications have analyzed the prognostic impact of ALDH1A1 expression. Hereby, the overexpression of ALDH1A1 has been controversially described as a dismal 18, good 29 and non-relevant 30 prognosticator.

## Materials and Methods

### Human tissue samples

The presented study included 69 chemonaive patients who underwent primary resection for intrahepatic cholangiocarcinoma at University Hospital Freiburg from 2000 to 2014. The study received approval from the local ethics committee of the Faculty of Medicine at the Albert-Ludwigs-Universität Freiburg (REF 21-1684).

All tissue specimens were processed for tumor diagnostics, involving standardized sectioning, 24-hour formalin fixation, paraffin embedding, cutting, hematoxylin-eosin staining, and review by experienced pathologists. After diagnosis, all slides were stored at the Institute for Surgical Pathology (ISP), University Center Freiburg, Germany. Diagnostic archive tissue specimens and corresponding slides were retrieved from the ISP archive and re-reviewed according to current UICC and WHO classifications. Next, all tissue specimens and corresponding clinicopathological data were pseudonymized. Clinicopathological data included TNM classification, WHO classification, R-status, gender, age at iCC onset, and patients' overall survival.

### TMA construction

Digitized pseudonymized H&E slides (PANNORAMIC® 1000-Scanner, Sysmex) were uploaded to a local server (Casecenter, Sysmex, Service Unit v. 2.0903111521). For each patient, three circular tumor regions of interest (ROIs), each 1.0 mm in diameter were annotated using the Caseviewer software (Sysmex, v. 2.3) by experienced pathologists. The tissue microarrays (TMAs) were constructed with the TMA Grand Master (Sysmex). After loading the microarrayer with donor and acceptor blocks, the TMA Grand Master identified each block's barcode labeling according to its annotated block pseudonym. The TMA Grand Master accessed the local server and matched the previously annotated and uploaded digital slides with the corresponding donor block. All slides were aligned with the photographed donor blocks and reviewed for accurate consistency. Next, annotated ROIs were transferred to the acceptor block. Considering TMA design, an asymmetric array for simple TMA orientation with 11 columns and 17 rows was chosen. To avoid corrupting batch effects, the acceptor TMA arrays were designed and transferred in a random fashion. Additionally, each TMA received on-slide control cores (placenta and tonsil, three each per TMA acceptor block).

After the transfer process's completion, the acceptor block was placed on top of a glass slide, heated to 37 °C for 60 minutes, and immediately cooled down on crushed ice for 10 minutes. This procedure was repeated twice.

### Immunohistochemistry

Immunohistochemical staining was performed with a commercially available primary antibody for Human Aldehyde Dehydrogenase 1-A1 (ALDH1A1; R&D Systems, mouse monoclonal Mouse IgG2B Clone # 703410 [MAB5869]) and the EnVision FLEX detection system (Dako, K8000). Tissue sections of 2µm thickness were cut (Leica RM2255 rotary microtome) and deparaffinized in the ISP. The staining protocol included the following steps: incubation with blocking reagent (EnVision FLEX Peroxidase Blocking Reagent, DAKO) for 10 minutes, ALDH1A1 staining with the afore mentioned primary antibody at a concentration of 2,5µg/ml (1/200), with mouse linker (EnVision Flex + Mouse Linker, DAKO) for 15 minutes, with horseradish peroxidase (EnVision FLEX/HRP, DAKO) for 20 minutes, with Envision FLEX DAB + Substrat Buffer (1:51) for 10 minutes and finally counterstained in hematoxylin.

**Figure 1:**
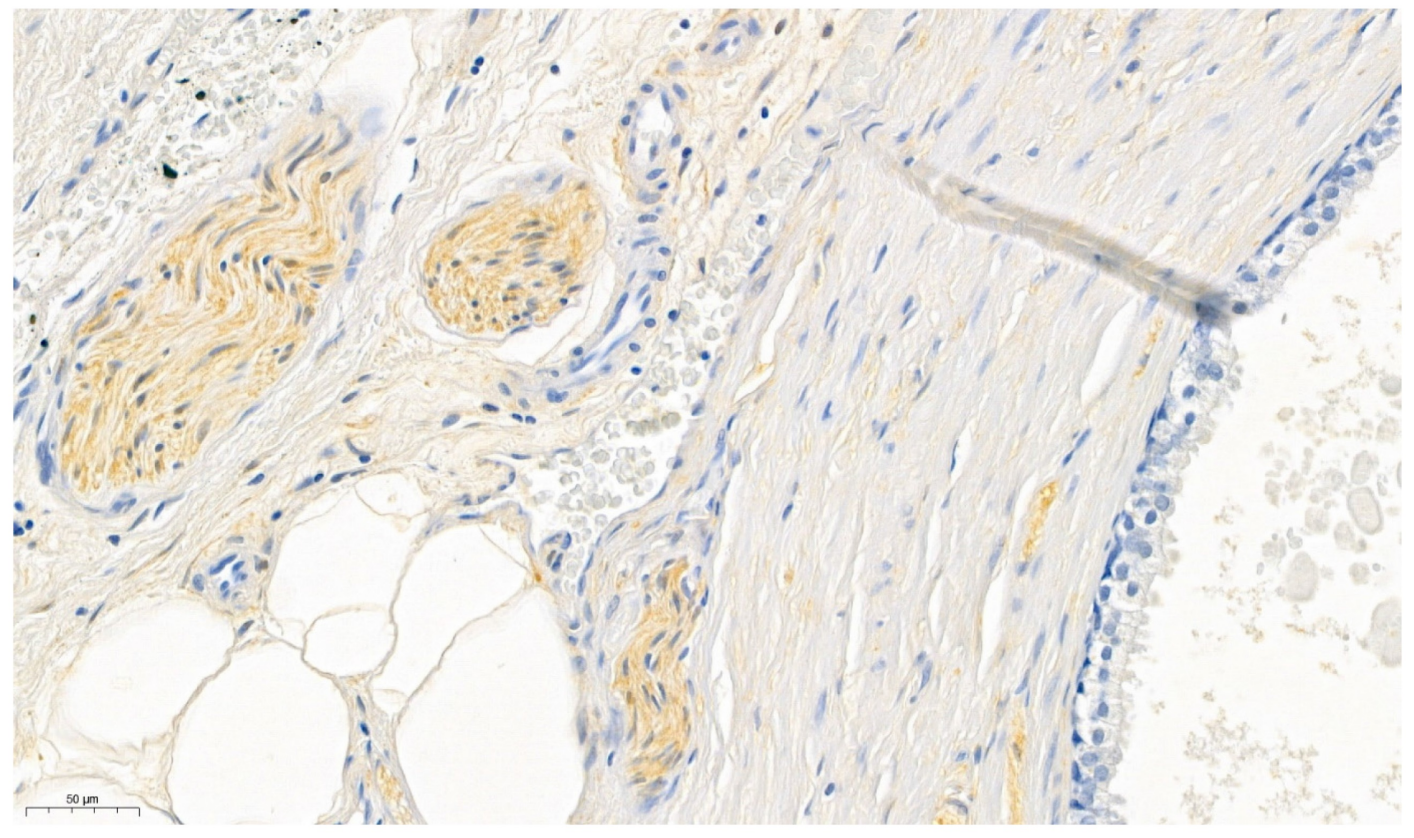
Prostate specimens were used for validating the ALDH1A1 antibody. The perinueral sheaths served as positive controls whereas the prostatic glands served as internal negative controls.

As positive control peripheral nerve bundles (in prostate specimen), known for its consistent expression of ALDH1A1 31, were used (Figure 1). The surrounding prostatic glands served as internal negative control 32. Furthermore omission of the primary antibody was conducted for negative control.

### Tissue microarray analysis

For digital image analysis, the immunohistochemically stained TMA slides were digitized (MIRAX) and imported into the open-source freeware QuPath (v. 0.3.0) 33. After applying the TMA dearrayer to identify tissue cores ("TMA core diameter": 1.2 mm, "Column labels": X1-X11, "Row labels": Y1-Y17, "Label Order": Row first, "Density threshold": 4, "Bound scale factor": 105), the TMA grid was adjusted. Missing cores or cores with prominent artifacts were discarded; inaccurately aligned core annotations were adjusted. A TMA map with patient identifiers was imported to enable correlation with clinical data. For proper stain separation, automated color deconvolution was applied on a representative region including strong hematoxylin and DAB staining, as well as an area of background.

Cells were detected and their cytoplasmic immunohistochemical staining was evaluated using the “Positive cell detection command” (“Detection image: Hematoxylin OD”, “Requested pixel size: 0.5 µm”, “Background radius: 0.0µm”, “Median filter radius: 0.0 µm”, “Sigma: 1.0 µm”, “Minimum area: 15.0 µm²”, “Maximum Area: 350.0 µm²”, “Threshold: 0.2”, “Max background intensity: 0.0”, “Split by shape: true”, “Exclude DAB (membrane staining): false”, “Cell Expansion: 5.0 µm”, “Include cell nucleus: true”, “Smooth boundaries: true”, “Make measurements: true”, “Threshold compartment: Cytoplasm: DAB OD mean”, “Threshold 1+: 0.075”, “Threshold 2+: 0.15”, “Threshold 3+: 0.35”, “Single threshold: false”).

**Figure 2:**
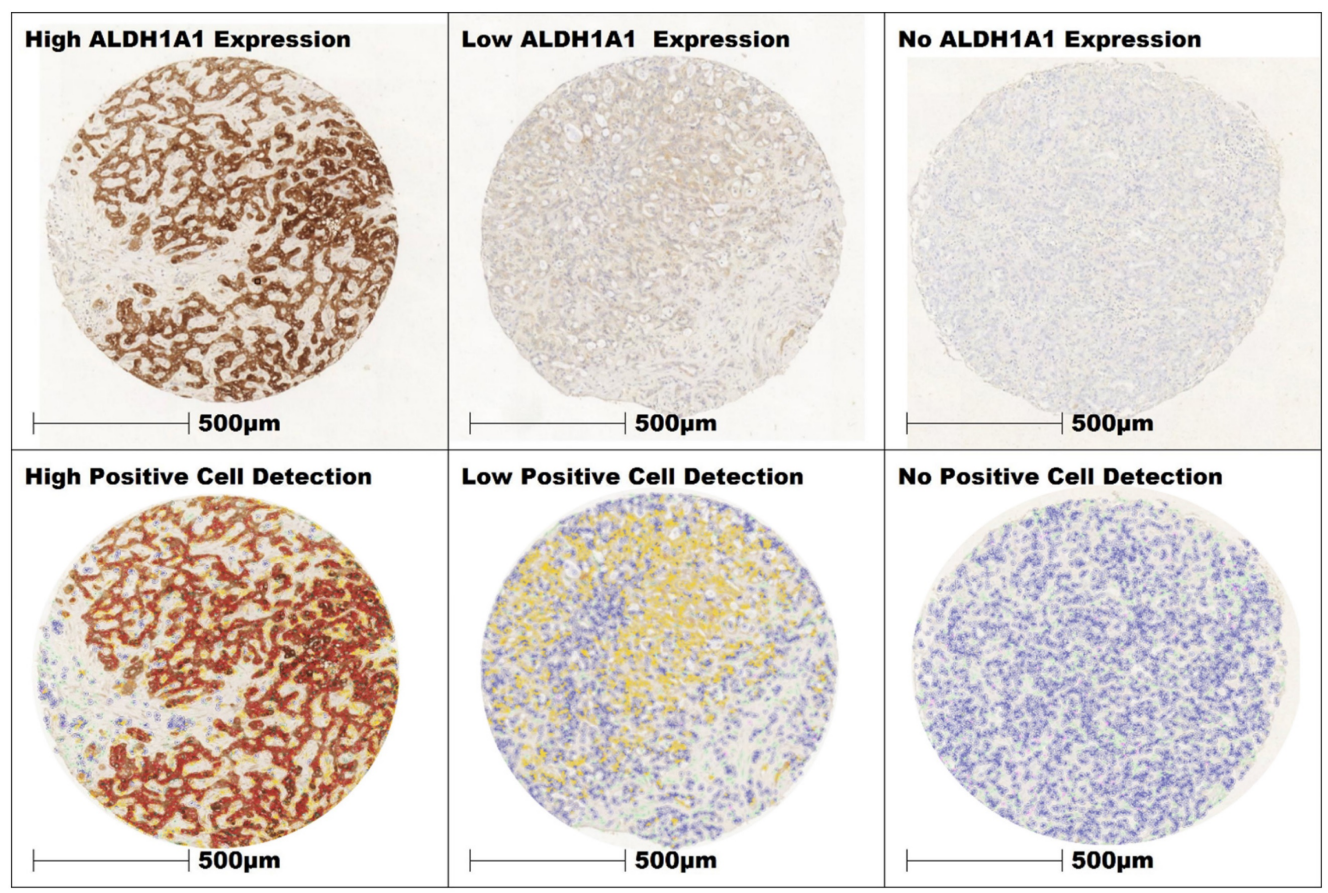
Three cores of iCC with high, low and no expression of ALDH1A1 in the tumor cells. The intensity of expression was color-coded in QuPath (red = 3+, orange = 2+, yellow = 1+, blue 0).

For tissue classification a total of 8570 cells with a cumulative area of 1.2 mm² were annotated as tumor, stroma, and “other” cells (e.g., immune-cell infiltrates, necrotic cells) separately (“Classify” > “Object classifier” > “Train object classifier”) throughout various TMA cores, incorporating all TMA slides. These manually classified cells served as training data for the classification model using the Random Tree machine learning algorithm. The model was trained on the extracted features of the training data to capture the patterns and relationships between these features and the target classes. The model evaluated the similarity of each cell's features with the known features of the training data and assigned them to the corresponding classes. The classifier was applied across all TMA cores. The resulting cell classification was visually evaluated by a pathologist on all cores for its discriminatory power and reliability in the assignment of tissue classes. Based on the review of the results, adjustments were made to improve performance by annotating misclassified cells. Through iterative adjustment and review, the cell classification model was continuously optimized (Figure 2). The resulting cell classification and staining intensity were re- examined and reviewed by pathologists for data consistency. The derived data for tumor and stroma cells of each core, including their respective patient identifiers, were then exported as a text file for further statistical analysis.

### Statistical analysis

All statistical analyses were performed with RStudio (RStudio, PBC, Boston, MA; desktop v. 1.4.1717), which offers a graphical user interface for R (The R Foundation for Statistical Computing, Vienna, Austria, v. 4.0.3). Median values, percentages of total, and estimated median overall survival were calculated as described in the descriptive statistics section (Table 2). For survival estimation, the survival (v. 3.2-13) and survminer (v. 0.4.9) packages in R were utilized. The Kaplan-Meier method was employed to estimate survival curves. The log-rank test was applied for analyzing differences in survival based on variables such as ALDH1A1 Expression, Age, Sex, pT- and pN-status, and lymph vessel invasion. To dichotomize ALDH1A1 expression and age, the "cutpointr" function from the cutpointr package (v. 1.1.2) was used to determine optimal cut-off values. This function determines the optimal threshold for splitting a continuous variable, such as biomarker expression, to help predict survival outcomes. It assesses various thresholds of the biomarker to find the cutpoint that optimizes a selected metric—typically the sum of sensitivity and specificity—relative to a binary outcome, like survival status 34. This allows for the classification of patients into high-risk and low-risk groups based on their biomarker levels.

Cox Proportional Hazards Models were applied to evaluate the impact of variables such as ALDH1A1 Expression, Age, Sex, T- and N-Classification, Grading, Blood and Lymph Vessel Invasion. For all statistical methods, the p-value for significance was defined as p ≤0.05.

## Results

### Clinicopathological parameters

In total, 69 patients (34 male (51%); 35 female (49%)) with a mean age of 64 years at the time of surgical resection (range 33 - 83) were included in the study.

In accordance with the UICC-Classification (8th version), 33 patients (48%) were classified as pT1, 18 patients (26%) as pT2, 13 patients (19%) as pT3, and 5 patients (7%) as pT4. The mean tumor size was 8.6 cm with a range from 2.5 - 22 cm. Tumor differentiation was graded as G1 in 7 cases (10%), G2 in 40 cases (58%), and G3 in 22 cases (32%). Lymphadenectomy was performed in 39 cases (56%), with evidence of lymph node metastasis in 14 cases (pN1; 20%). No lymph node metastasis was found in 25 cases (pN0; 36%). Due to the extended inclusion period of the study, Lymphadenectomy was not consistently performed as a standard procedure. Lymph vessel invasion was histologically identified in 20 cases (L1; 29%), blood vessel invasion in 15 (V1; 22%), and perineural sheath invasion in 22 cases (Pn1; 32%). A histologically complete resection (R0) was achieved in 53 patients (77%), and an incomplete resection with residual disease in 16 patients (R1; 23%).

### Descriptive statistics: ALDH1A1 expression in tumor and stromal-cells

ALDH1A1 expression in both tumor and stromal cells was assessed for all patients, revealing a skewed distribution. The median expression of ALDH1A1 in tumor cells was 90.53%, with a range of 0.00 to 99.75%. Tumor-associated stromal cells exhibited ALDH1A1 expression ranging from 0.00 to 99.45%, with a median of 70.45%.

In the female population, the median ALDH1A1 expression in tumor cells was 92.56%, with values ranging from 2.03 to 99.66%. The associated stromal cells had a median expression of 77.87%, with a range of 1.42 to 99.46%.

In the male subgroup, the median ALDH1A1 expression in tumor cells was 81.51%, with a range from 0.00 to 99.75%, and 63.34% in tumor-associated stromal cells, with a range from 0.00 to 96.84%.

Patients were dichotomized by a cutoff into high- and low-ALDH1A1 expression groups, as described above. The cutoffs for tumor- and stromal-cell expression of ALDH1A1 were set at 94.92% and 88.45%, respectively.

**Figure 3:**
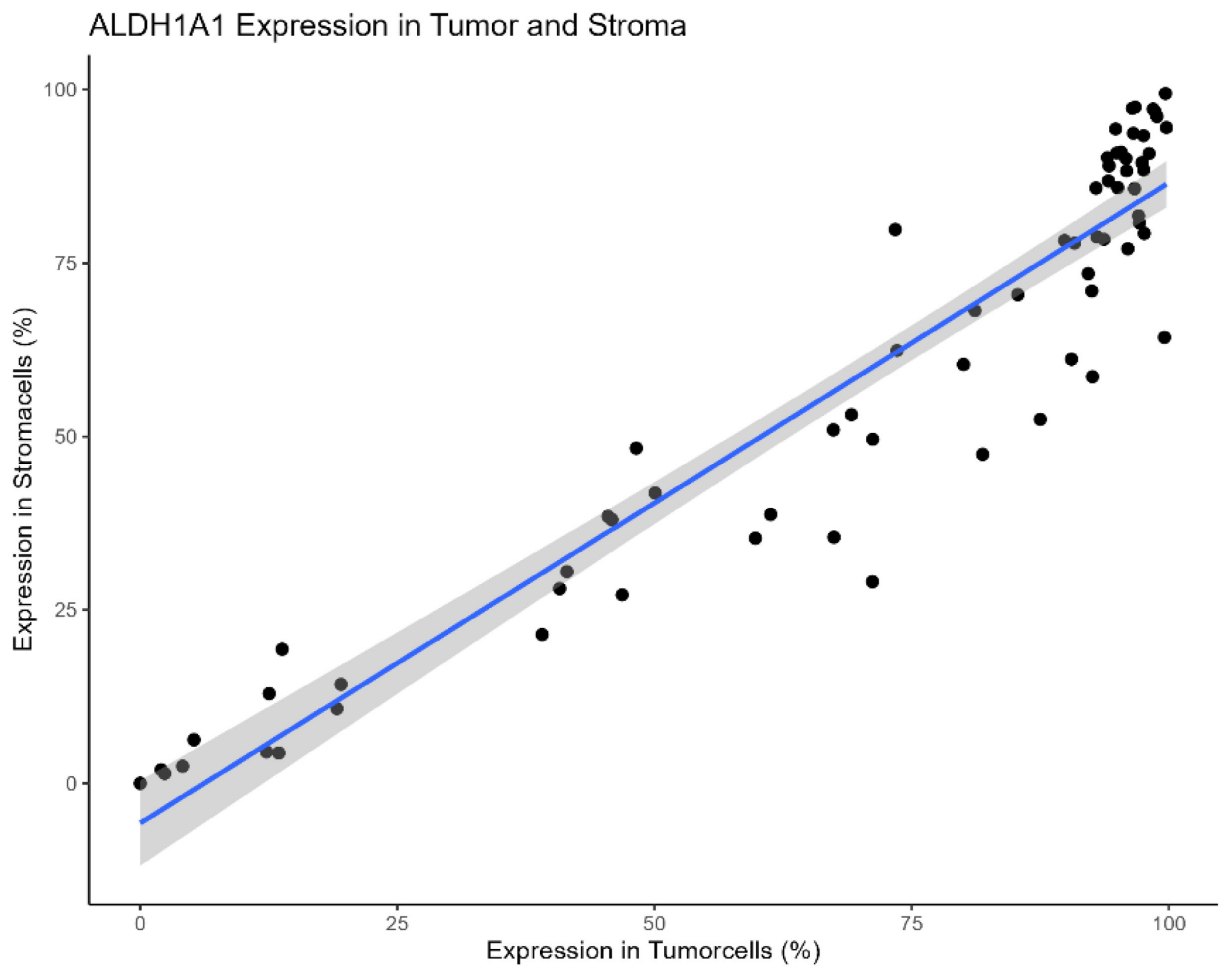
Scatter-Plot of ALDH1A1 Expression in tumor and stromacells of individual patients.

The Pearson´s Chi-squared test showed a significant correlation of ALDH1A1 Expression within tumor and its surrounding stroma cells (p = < 0.001, Table 1, Figure 3).

### Survival Analysis

The estimated mean patient overall survival was 48.26 months (range 2 - 180 months), with a median overall survival of 33 months. At the time of the last follow-up, 58 patients (84%) had died. The remaining 11 patients (16%) were censored.

In the Log-rank Analysis for overall survival and the pathological parameters, significantly shortened overall survival was observed for patients with local lymph node metastasis and lymph vessel invasion (p < 0.0001 each). Similarly, an advanced tumor class (> pT1; p = 0.03), incomplete surgical resection (R1; p = 0.013), and blood vessel invasion (V1; p = 0.048) significantly shortened overall survival. Other clinicopathological parameters did not show a significant impact on overall survival in this study (Sex, Age, Perineural Sheath Invasion (Pn), Tumor Grade).

**Figure 4:**
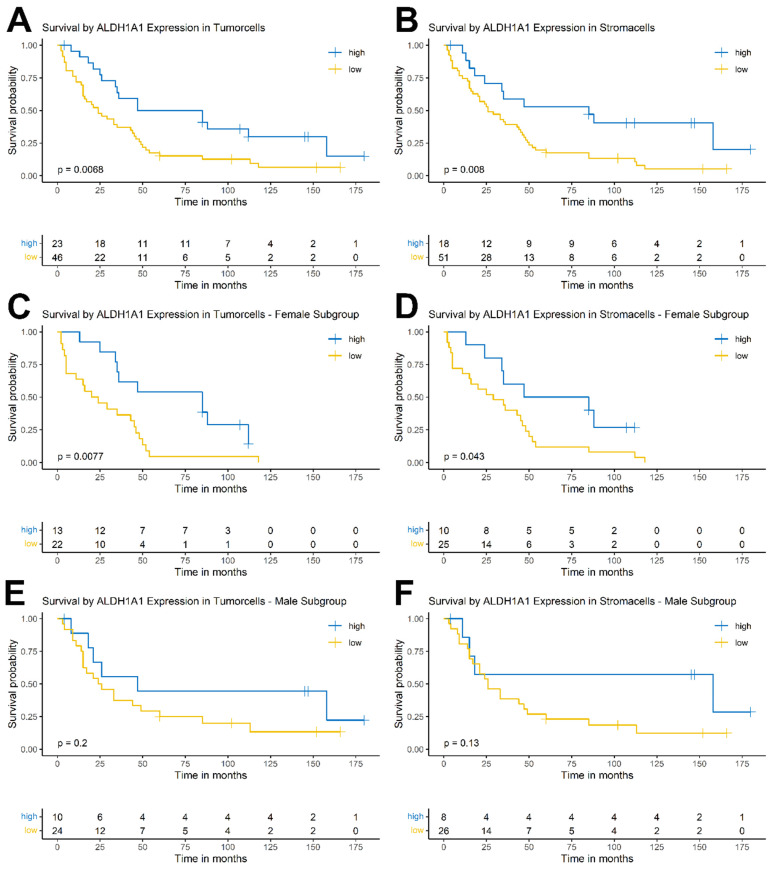
Kaplan-Meier survival curves of ALDH1A1 expression in tumor and surrounding stromal cells of the whole cohort (A, B) as well as in the female (C, D) and male (E, F) subgroup.

In both tumor and stromal cells, high ALDH1A1 expression correlated with significantly longer patient overall survival (ALDH1A1 expression in tumor p = 0.0068; ALDH1A1 expression in stroma p = 0.008) (Figure 4 A, B)).

Next, patients were stratified by sex using the same cutoffs. This split showed significantly prolonged overall survival in the female subpopulation for high ALDH1A1 expression in tumor (p = 0.0077) and stromal cells (p = 0.043) (Figure 4 C, D). There were no significant results in the male subpopulation for expression in tumor or stromal cells (p = 0.2; p = 0.13, respectively) (Figure 4 E, F).

### Analysis of the hazard ratio using the Cox-regression (Table 2 and Table 3)

Univariate Cox regression model showed prognostic significance for ALDH1A1 expression in both tumor and stromal cells. Low ALDH1A1 expression correlated with a dismal survival (tumor: HR = 2.19; CI [1.22, 3.93]; p = 0.008; stroma: HR = 2.4; CI [1.23, 4.67]; p = 0.01). Further significant prognosticators included T-Classification (pT3: HR = 2.36; CI [1.17, 4.75]; p = 0.016), lymph node involvement (pN1: HR = 3.47; CI [1.66, 7.26]; p < 0.001), perineural sheath invasion (HR = 1.77; CI [1.01, 3.11]; p = 0.046), lymph vessel invasion (HR = 3.05; CI [1.7, 5.49]; p < 0.001), blood vessel invasion (HR = 1.89; CI [1.02, 3.49]; p = 0.042), and incomplete surgical resection (HR = 2.66; CI [1.44, 4.89]; p = 0.002). Tumor-grade, patient age, or sex did not show a significant impact on the hazard ratio.

Due to the co-dependency of ALDH1A1 expression in tumor and stromacells, as shown in Table 1 and Figure 3, multivariable Cox regression models were performed separately for each, encompassing all other univariably significant variables (pT, pN, Pn, L, V, Residual Disease) each yielding significant hazard ratios for ALDH1A1 expression. As in the univariable analysis low ALDH1A1 expression correlated with an increased risk of death (tumor: HR = 1.95; CI [1.04, 3.66]; p = 0.036; stroma: HR = 2.23; CI [1.11, 4.51]; p = 0.025).

In the female subpopulation, low ALDH1A1 in either tumor or stroma correlated with an increased risk of death (tumor: HR = 2.8; CI [1.27, 6.17]; p = 0.01; stroma: HR = 2.36; CI [1.0, 5.55]; p = 0.049). Furthermore T-Classification (pT2: HR = 2.7; CI [1.16, 6.24]; p = 0.021), lymph node involvement (pN1: HR = 4.73; CI [1.65, 13.6]; p = 0.004), lymph vessel invasion (HR = 8.16; CI [2.93, 22.8]; p < 0.001), blood vessel invasion (HR = 3.72; CI [1.46, 9.45]; p = 0.006), and incomplete surgical resection (HR = 3.92; CI [1.65, 9.3]; p = 0.002). Tumor-grade, patient age, and perineural sheath invasion did not show a significant impact on the hazard ratio. ALDH1A1 expression did not show an independently significant impact in the multivariable regression models of the female subpopulation.

The Cox regression of the male subpopulation did not show a significant impact of ALDH1A1 Expression on risk of death, neither in the uni- nor on the multivariable model.

## Discussion

Recent advancements in oncological treatments have underscored the importance of biomarkers. These biomarkers are broadly categorized into predictive, such as specific receptors in breast cancer and EGFR in metastatic non-small cell lung cancer 35 and prognostic, which indicate disease progression, recurrence, or mortality 36.

In iCC, patient outcomes are predominantly determined by the tumor stage at diagnosis. Current biomarkers for iCC, like CA19-9, CA 125, and CEA, are limited by their specificity and sensitivity, confining their use mainly to monitoring disease progression rather than offering diagnostic or therapeutic insights 37-39. This highlights the need for more reliable prognostic or predictive biomarkers in iCC clinical practice.

ALDH1A1's prognostic significance varies across different cancers. Ding *et al.* highlighted a negative prognostic impact associated with high ALDH1A1 expression in cholangiocellular carcinoma, disregarding the anatomical cancer origin (intra- and extrahepatic cholangiocarcinoma). Furthermore their review focused on *in vitro* studies and not *in vivo* data / comprising patients outcome 40. In iCC, research linking ALDH1A1 to patient overall survival has been inconsistent, potentially due to methodological variations, including the use of immunohistochemistry on whole slides versus TMAs and manual versus AI-based evaluations 18,30. Additionally, differing etiologies between Asian and Western patients, such as liver fluke infections and chronic hepatitis B and C in Asia versus primary sclerosing cholangitis and metabolic syndrome in the West, might lead to different impacts of ALDH1A1 expression on survival in studies performed 41,42. Furthermore, the heterogeneous nature of iCC and its diverse genetic and environmental influences might contribute to these discrepancies 43. Finally, the dual role of ALDH1A1 in both tumor cells and the tumor microenvironment might lead to complex interactions that are not yet fully understood 44,45.

Our study utilized a TMA-based approach with AI for objective, user-independent evaluation, minimizing staining variability 46 and reducing interobserver variability. The importance of the tumor microenvironment in cancer progression is increasingly recognized in oncological research 47-53.

Interestingly, ALDH1A1 expression in stromal cells has been largely overlooked. Only a few isolated studies, particularly in breast cancer, have explored its role in the TME and its impact on tumorigenesis.

Our findings reveal that ALDH1A1 overexpression in tumor cells correlates with prolonged patient survival in iCC, contradicting its typical association with higher tumorigenicity 54,55, and cancer stem cells 56. Our data also indicated a significant correlation between ALDH1A1 overexpression in stromal cells and improved patient outcomes, consistent with previous reports in iCCs 29 and other primary liver cancers 27,57-59.

Furthermore, we investigated ALDH1A1's impact on patient outcomes based on gender, observing a significant effect in female patients. Cox regression analysis indicated significant hazard ratios for ALDH1A1 expression in both tumor and stromal cells, in univariable as well as multivariable models. Given that intrahepatic cholangiocellular carcinoma falls under the category of rare diseases, resulting in a naturally low patient count, there is a pressing need for further studies to investigate potential gender-specific biological mechanisms underlying ALDH1A1 expression in iCC.

The immunohistochemical detection of ALDH1A1 is straightforward and cost-effective, suggesting its potential as a reliable prognostic marker in iCC. However, the current lack of effective treatment options for iCC means that even a favorable prognosis does not significantly alter therapeutic strategies. The possibility of ALDH1A1 serving as a predictive marker for targeted therapies remains an area for future exploration 60,61.

## Conclusion

ALDH1A1 overexpression in both tumor and stromal cells has been shown to favorably impact overall survival in iCC. However, these results need to be validated in larger patient cohorts. If validated, ALDH1A1 could become a significant prognostic biomarker, aiding future patient management. The study's findings on ALDH1A1, particularly its varying impact based on gender, provide new insights. The favorable prognostic impact of high ALDH1A1 expression in tumor cells aligns with results from hepatocellular carcinoma studies, suggesting a broader relevance across primary liver cancers. This study's methodology, focusing on the tumor microenvironment and employing advanced techniques like AI-supported evaluation, underscores the evolving landscape of oncological research and the potential for new therapeutic strategies in managing devastating conditions like iCC.

## Figures and Tables

**Table 1:**
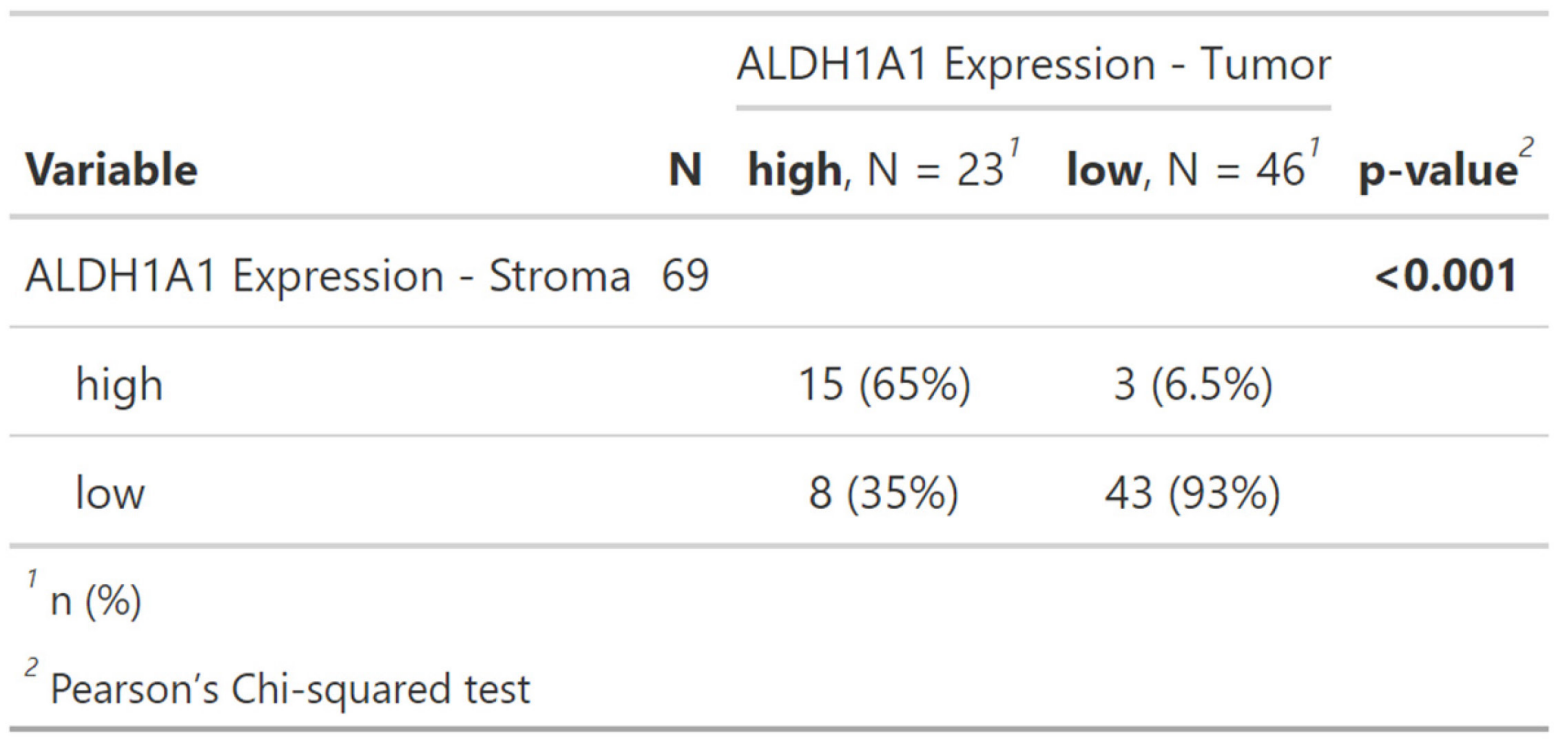
The Pearson´s Chi-squared test showed a significant correlation of ALDH1A1 Expression within tumor and its surrounding stroma cells (p = < 0.001).

**Table 2:**
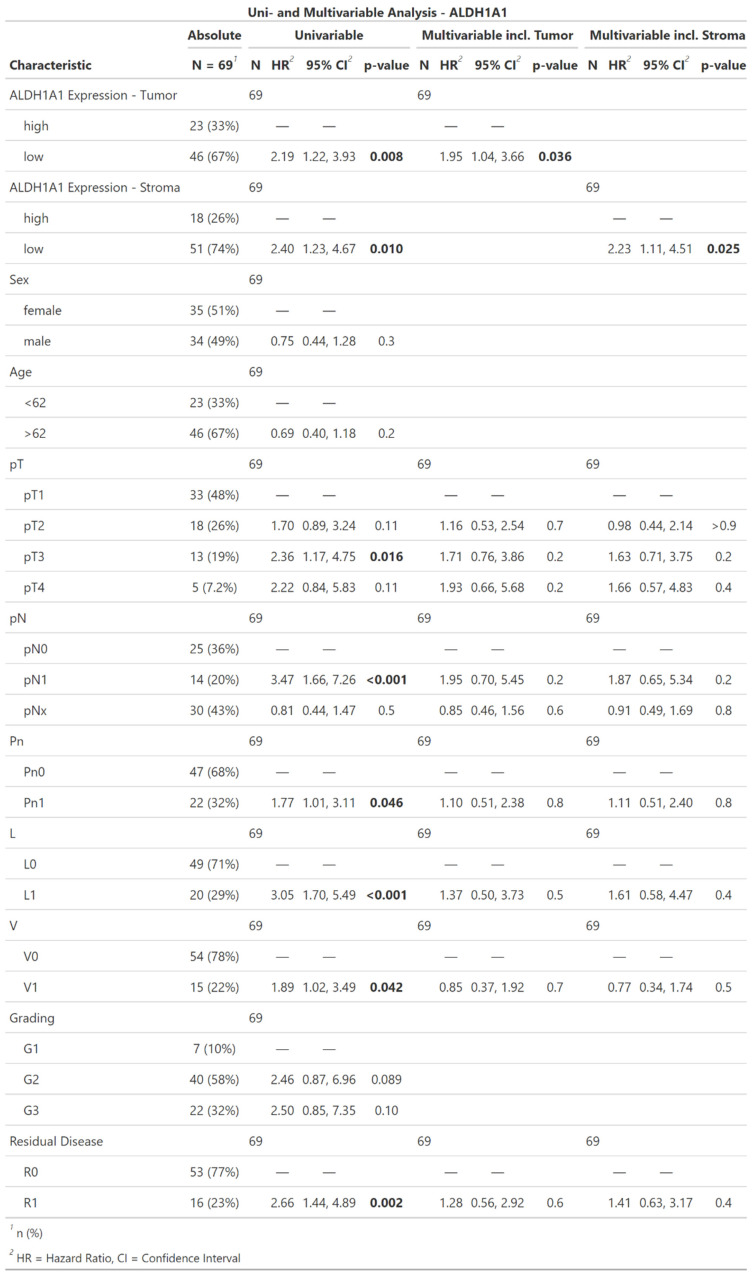
Descriptive statistics, univariable and multivariable Cox regression of ALDH1A1 expression in iCC. Significant results are highlighted in bold.

**Table 3:**
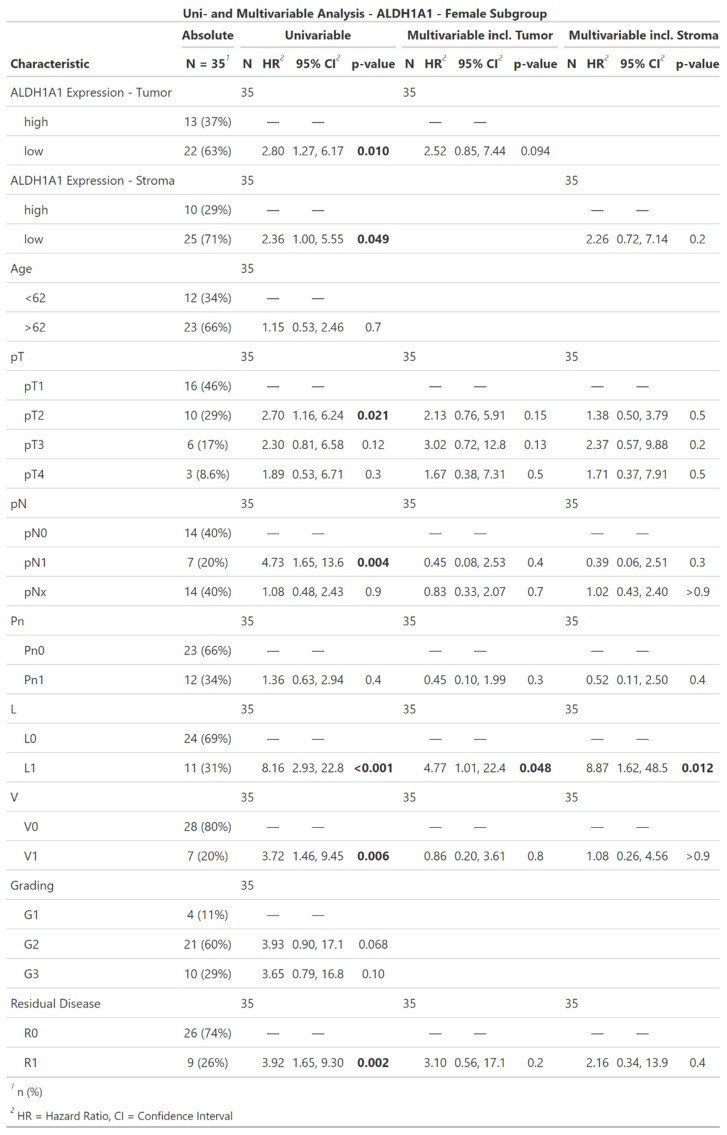
Descriptive statistics, univariable and multivariable Cox regression of ALDH1A1 expression in iCC within the female subpopulation. Significant results are highlighted in bold.
